# Phytochemical Constitution, Anti-Inflammation, Anti-Androgen, and Hair Growth-Promoting Potential of Shallot (*Allium ascalonicum* L.) Extract

**DOI:** 10.3390/plants11111499

**Published:** 2022-06-02

**Authors:** Warintorn Ruksiriwanich, Chiranan Khantham, Anurak Muangsanguan, Chuda Chittasupho, Pornchai Rachtanapun, Kittisak Jantanasakulwong, Yuthana Phimolsiripol, Sarana Rose Sommano, Korawan Sringarm, Emilia Ferrer, Francisco J. Barba

**Affiliations:** 1 Department of Pharmaceutical Sciences, Faculty of Pharmacy, Chiang Mai University, Chiang Mai 50200, Thailand; ckhantham@gmail.com (C.K.); anurak_m@cmu.ac.th (A.M.); chuda.c@cmu.ac.th (C.C.); 2 Cluster of Research and Development of Pharmaceutical and Natural Products Innovation for Human or Animal, Chiang Mai University, Chiang Mai 50200, Thailand; sarana.s@cmu.ac.th (S.R.S.); korawan.s@cmu.ac.th (K.S.); 3 Cluster of Agro Bio-Circular-Green Industry, Faculty of Agro-Industry, Chiang Mai University, Chiang Mai 50100, Thailand; pornchai.r@cmu.ac.th (P.R.); kittisak.jan@cmu.ac.th (K.J.); yuthana.p@cmu.ac.th (Y.P.); 4 School of Agro-Industry, Faculty of Agro-Industry, Chiang Mai University, Chiang Mai 50100, Thailand; 5 Department of Plant and Soil Sciences, Faculty of Agriculture, Chiang Mai University, Chiang Mai 50200, Thailand; 6 Department of Animal and Aquatic Sciences, Faculty of Agriculture, Chiang Mai University, Chiang Mai 50200, Thailand; 7 Department of Preventive Medicine and Public Health, Food Science, Toxicology and Forensic Medicine, Faculty of Pharmacy, University of Valencia, 46100 Valencia, Spain; emilia.ferrer@uv.es (E.F.); francisco.barba@uv.es (F.J.B.)

**Keywords:** androgenetic alopecia, anti-hair loss, hair growth promotion, shallot, *Allium ascalonicum*, anti-inflammatory, 5α-reductase, *SRD5A2*, Wnt/β-catenin

## Abstract

In Thai folklore wisdom, shallot (*Allium ascalonicum* L.) was applied as a traditional herbal medicine for hair growth promotion with no scientific evidence. Androgenetic alopecia (AGA) is a progressive hair loss caused by multiple factors, including androgen hormones, inflammation, and oxidative stress. Conventional medicines (finasteride, dutasteride, corticosteroids, and minoxidil) have been used with limited therapeutic efficacy and unpleasant side effects. In this study, we aimed to give the first estimation of bioactive compounds in shallot extract and evaluate the hair growth-promoting activities regarding anti-inflammatory and gene expression modulation involving androgen, Wnt/β-catenin, sonic hedgehog, and angiogenesis pathways. The results reveal that phenolic compounds (quercetin, rosmarinic, and *p*-coumaric acids) are the major constituents of the methanolic shallot extract. Compared with the lipopolysaccharide-stimulated control group (2.68 ± 0.13 µM), nitric oxide production was remarkably diminished by shallot extract (0.55 ± 0.06 µM). Shallot extract improves hair growth promotion activity, as reflected by the downregulation of the androgen gene expression (*SRD5A1* and *SRD5A2)* and the upregulation of the genes associated with Wnt/β-catenin (*CTNNB1*), sonic hedgehog (*SHH*, *SMO*, and *GIL1*), and angiogenesis (*VEGF*) pathways. These findings disclose the new insights of shallot extract on hair growth promotions. Shallot extract could be further developed as nutraceutical, nutricosmetic, and cosmeceutical preparations for AGA treatment.

## 1. Introduction

Shallot (*Allium ascalonicum* L.) from the Alliaceae family is a valuable horticultural spice that originated in Southeast Asia. Shallot bulbs have been widely utilized as a major component in Asian diets and traditional herbal medicines in Thailand and India [1]. This plant possesses various properties, including anti-cancer, anti-diabetes, antimicrobial, anti-inflammatory, and antioxidant activities [1,2,3]. In addition, several health benefits of shallot have been reported, including wound healing and maintaining healthy skin and hair [1,4]. The most abundant components of shallots are phenolic compounds, saponins, and carbohydrates [5,6]. In addition, fresh shallot bulbs have been used in traditional Thai folklore to treat bacterial skin infections, tinea capitis, and hair loss.

Androgenetic alopecia (AGA) is a chronic hair loss, characterized by hair follicle miniaturization and perifollicular inflammation [7]. The pathogenesis of AGA is extensively influenced by genetic factors and androgens [8]. Androgen-mediated follicular miniaturization is the most elucidated pathogenesis of AGA [7,8]. Testosterone is metabolized to dihydrotestosterone (DHT)—a potent androgen—by steroid 5α-reductases [8]. Additionally, a histological evaluation of AGA’s scalp found perifollicular inflammation [9]. Another study proposed that nitric oxide-mediated perifollicular inflammation arose in response to DHT level [10]. Antiandrogenic medicines (finasteride, dutasteride, and spironolactone), anti-inflammatory corticosteroids, and the anti-seborrheic tretinoin have been used to attenuate AGA progression [11]. However, those have shown limited efficacy and unsatisfactory side effects [11,12]. Presumably, other factors exist in the gradual process of hair follicle miniaturization besides the androgenic effect [9].

The hair growth cycle consists of four phases: anagen (growth phase), catagen (regression phase), telogen (resting phase), and exogen (shedding phase) [13,14]. Multiple signaling pathways involve the transition between these phases, including Wnt/β-catenin, sonic hedgehog, and angiogenesis signaling pathways [9,13]. The Wnt/β-catenin and sonic hedgehog signaling pathways involve the differentiation and proliferation of hair follicle cells [15,16]. Vascular endothelial growth factor (VEGF) has been reported to be implicated in the angiogenesis in the anagen phase and facilitate the supply of oxygen and nutrients to hair follicles [13]. Minoxidil has been widely used to stimulate hair growth in males and females with AGA due to its ability to dilate the blood vessels in the follicle [11]. The synthetic medicines targeting Wnt/β-catenin and sonic hedgehog signaling pathways have not been implemented for the treatment of hair loss [13,15].

Hair follicle inflammation has been confirmed as a possible factor in the pathogenesis of AGA [17]. The nitric oxide (NO) level, which mediates inflammatory reaction in hair follicles, was increased in response to the DHT level [10]. In addition, it has been revealed that serum samples of AGA contained a higher level of NO than the control group [18]. The ultraviolet radiation stimulated NO production in keratinocytes of hair follicles. Consequently, the proinflammatory cytokines were released, and the recruitment of immune cells was facilitated, resulting in the damage of hair roots [19].

Conventional medicines for AGA have been shown to have several side effects, such as scalp dryness, skin irritation, erectile dysfunction, and testicular pain [12]. These limitations contribute to the reduction in individual compliance with hair loss treatment. Since AGA requires long-term treatment, alternative treatments and natural herbal medicines have gained attention due to their advantages, including fewer side effects, a broad spectrum of hair growth-promoting activities, and affordable prices [13,20]. 

Currently, no strongly scientific evidence that supports the beneficial effects of shallot on AGA has been established. Furthermore, the effects of shallot on hair growth regulation at the cellular level have not been elucidated. The conversion of fresh shallots to concentrate extract might create a new perspective for developing nutraceuticals, nutricosmetics, and cosmeceuticals containing shallot extract for anti-hair loss. Considering all above reasons, with this first study, we aimed to estimate the bioactive compounds of shallot extract and investigate the anti-inflammatory activity and gene expression regulation involving androgen, Wnt/β-catenin, sonic hedgehog, and angiogenesis signaling pathways for AGA treatment.

## 2. Results

### 2.1. Extraction Yield and Bioactive Compound Estimation

**Figure 1:**
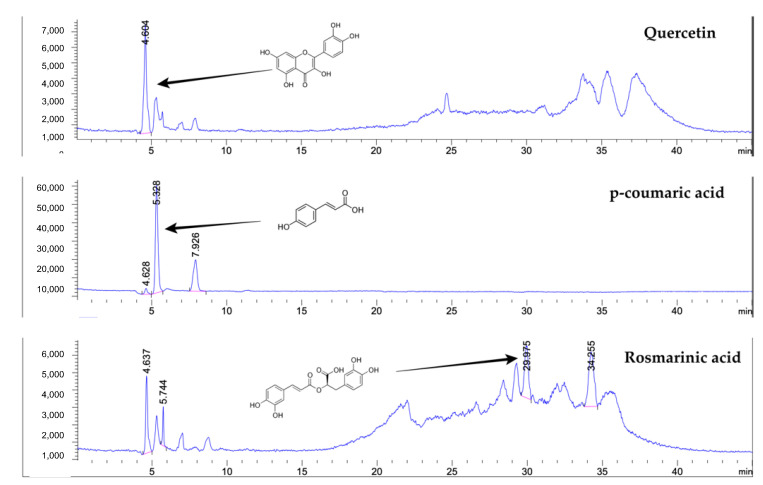
Chromatogram of bioactive compounds in shallot extract analyzed by liquid chromatography–mass spectrometry (LC-MS).

The physical appearance of shallot extract was a non-greasy paste with pink color. The extraction yield was 9.11 ± 0.23% *w*/*w* based on dry material. As shown in Table 1 and Figure 1, the most abundant bioactive compound in shallot extract was total phenolic content (4.96 ± 0.42 GAE/g), followed by contents of proteins, polysaccharides, and flavonoids. *p*-Coumaric acid, rosmarinic acid, and quercetin were the major phenolic compounds in shallot extract.

### 2.2. Cell Viability

**Figure 2:**
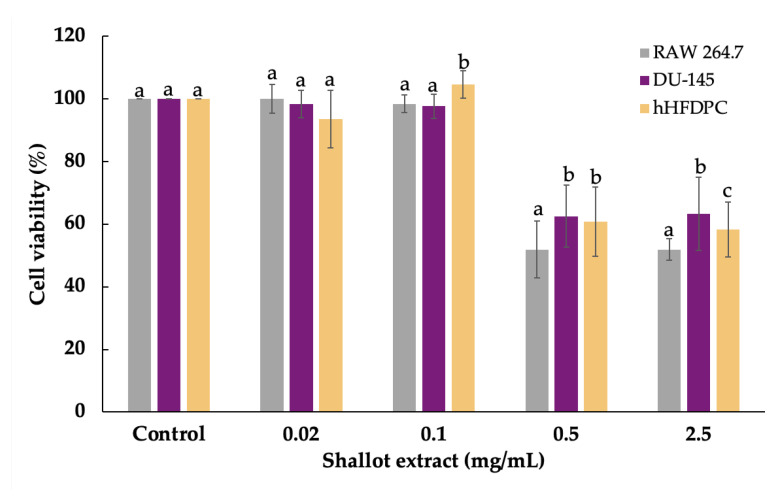
Cell viability of RAW 264.7 macrophage cells (RAW 264.7), DU-145 human prostate cancer cells (DU-145), and human hair follicle dermal papilla cells (hHFDPC) after shallot extract treatment for 24 h with different concentrations (0.02 to 2.5 mg/mL) was determined by sulforhodamine B (SRB) assay. Different letters (a, b, and c) indicate statistical differences (*p*-value < 0.05) in the cell viability of each concentration.

In order to evaluate the anti-inflammation and effects on gene expression profiling of shallot extract, the viability of cells used in this study, namely DU-145 and hHFDPC, was assessed by the sulforhodamine B (SRB) assay [21]. In Figure 2, shallot extract at the concentration above 0.5 mg/mL showed cytotoxicity and significantly decreased viability of all types of cells compared to corresponding solvent-treated control groups. The highest concentration of shallot extract (0.1 mg/mL) that gave the viability of RAW 264.7 cells above 80% was classified as a non-toxicity concentration and selected for all experiments [22].

### 2.3. Anti-Inflammatory Activity

**Figure 3:**
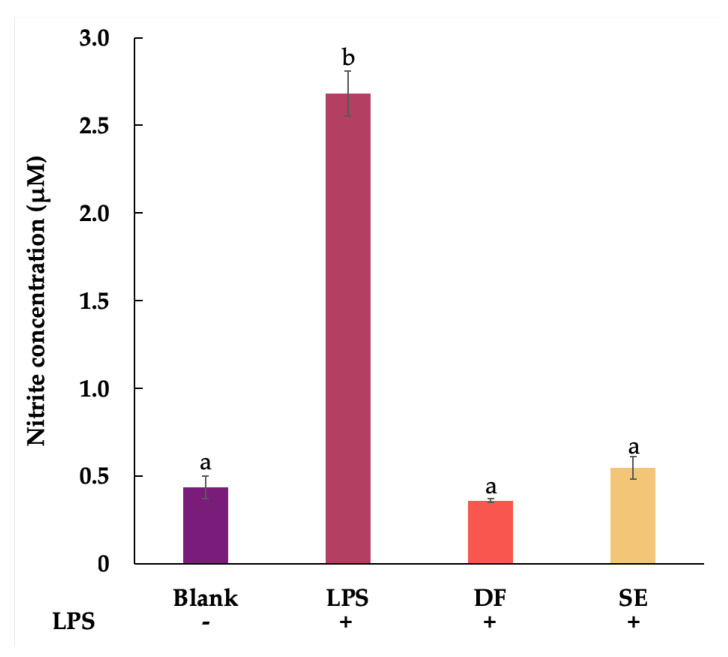
Effects of shallot extract (SE) and diclofenac sodium (DF) at the same concentration of 0.1 mg/mL on nitrite production in the lipopolysaccharide (LPS)-stimulated RAW 264.7 murine macrophages for 24 h compared to solvent-treated control without LPS (blank) and LPS-stimulated control (+LPS). Different letters (a and b) indicate statistical significance (*p* < 0.05) in comparison to +LPS and DF.

Therefore, diclofenac sodium (DF) at the same concentration (0.1 mg/mL) with no toxicity was selected to compare the inhibitory effect on NO production. The level of accumulated nitrite, which is the stable metabolite of NO, was indirectly quantified. In Figure 3, the concentration of nitrite in the lipopolysaccharide (LPS)-stimulated control group (2.68 ± 0.13 µM) explicitly increased compared with a solvent-pretreated group (0.43 ± 0.06 µM). The pretreatment with shallot extract significantly decreased the nitrite production to 0.55 ± 0.06 µM compared with the LPS-stimulated group (*p* < 0.05). Moreover, the NO inhibition of the DF-pretreated group (0.36 ± 0.01 µM) was significantly comparable to shallot extract.

### 2.4. Effect of Shallot Extract on Expressions of Genes Associated with Androgenetic Alopecia

**Figure 4:**
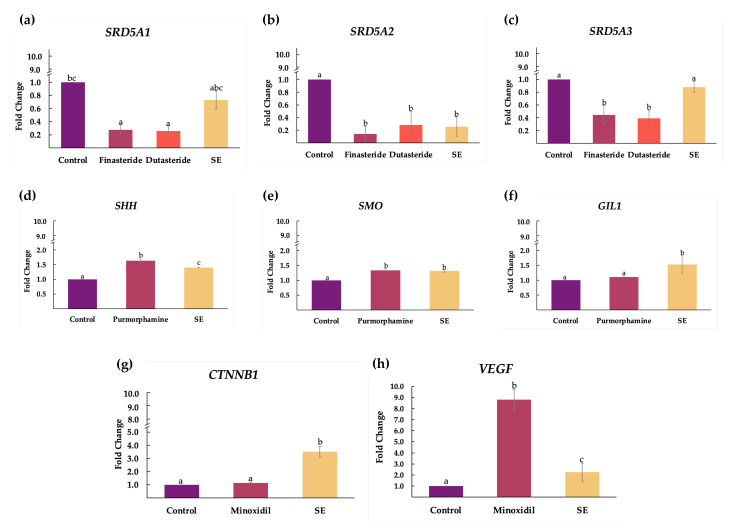
Effect of shallot extract (SE, 0.1 mg/mL) on expressions of genes associated with androgenetic alopecia: (**a**) *SRD5A1*; (**b**) *SRD5A2*; (**c**) *SRD5A3*; (**d**) *SHH*; (**e**) *SMO*; (**f**) *GIL1*; (**g**) *CTNNB1*; (**h**) *VEGF*. DU-145 human prostate cancer cells (DU-145) were used to observe the expressions of genes in the androgen pathway (*SRD5A* genes), whereas human hair follicle dermal papilla cells (hHFDPC) were used to study the remaining pathways. Different letters (a, b, and c) indicate statistical significance (*p* < 0.05) in comparison to control, finasteride (0.1 mg/mL), dutasteride (0.1 mg/mL), purmorphamine (0.1 mg/mL), and minoxidil (0.1 mg/mL).

In this study, we evaluated the regulatory effect of shallot extract (0.1 mg/mL) on mRNA expressions of genes associated with the pathogenesis of AGA, including androgen pathway (*SRD5A1*, *SRD5A2*, and *SRD5A3*), sonic hedgehog pathway (*SHH*, *SMO* and *GIL1*), Wnt/β-catenin pathway (*CTNNB1*), and VEGF signaling pathway (*VEGF*). The reference standard compounds, including finasteride, dutasteride, purmorphamine, and minoxidil, were used at the same concentration of 0.1 mg/mL in all experiments. The results are illustrated in Figure 4.

Steroid 5α-reductase types 1, 2, and 3 were encoded by *SRD5A1*, *SRD5A2*, and *SRD5A3*. Remarkably, the expressions of *SRD5A1* (fold change of 0.73 ± 0.14) and *SRD5A2* (fold change of 0.25 ± 0.16) were significantly suppressed in the shallot extract group compared with the control group (Figure 4a,b). There were no significant differences between shallot extract and the standard drugs (finasteride and dutasteride) regarding *SRD5A2* suppression (Figure 4b). However, the reduction in *SRD5A3* expression was not observed in shallot extract.

The molecules in sonic hedgehog pathways, which are sonic hedgehog (shh), smoothened (SMO), and GLI family zinc finger 1 (GIL1), were encoded by *SHH*, *SMO*, and *GIL1*, respectively. Compared with the control group, treatment with shallot extract upregulated the expressions of *SHH* (Figure 4d), *SMO* (Figure 4e), and *GIL1* (Figure 4f) in hHFDPC. In addition, the expression of *GIL1* markedly increased with a fold change of 1.53 ± 0.29, compared with the purmorphamine and control groups.

The gene encoding β-catenin is *CTNNB1.* In Figure 4g, the mRNA level of *CTNNB1* in the treatment of shallot extract in hHFDPC distinctly elevated with a fold change of 3.51 ± 0.41, compared with the minoxidil (1.15 ± 0.04) and control groups. On the other hand, minoxidil induced the upregulation of *VEGF* (fold change of 8.79 ± 0.96), as is evident in Figure 4h. *VEGF* expression was moderately upregulated with a fold change of 2.25 ± 0.82 in hHFDPC treated with shallot extract. 

## 3. Discussion

In this present study, phenolic compounds have been identified as the major compounds of methanolic shallot extract. Additionally, *p*-coumaric acid, quercetin, and rosmarinic acid were detected. Shallot extract diminished the NO production and secretion, contributing to anti-inflammatory activity. Shallot extract suppressed the expressions of *SRD5A1* and *SRD5A2* in DU-145 cell lines, whereas the expressions of genes associated with hair growth activation (*SHH*, *SMO*, *GIL1*, *CTNNB1*, and *VEGF*) were upregulated in hHFDPC. These findings demonstrate that shallot extract possesses hair growth-promoting effects through inhibiting inflammatory and androgen pathways. Wnt/β-catenin, sonic hedgehog, and VEGF pathways were also activated.

The disturbance of hair cycles leads to the elongation of resting phase, which contributes to AGA. Furthermore, it has been verified that progressive hair follicle miniaturization involves anagen shortening and premature catagen entry [9,23]. Since the anagen phase governs hair length, terminal hair eventually transforms to vellus hair in AGA [23]. Specialized mesenchymal cells, hHFDPC, play a vital role in hair follicle development and hair growth by supporting multi-potent stem cells, cytokines, growth factors, and nutrients [24]. Synchronized intercellular signaling cascades of hHFDPC and other adjacent cells implicate the hair follicle’s formation, maintenance, and homeostasis [7,23,25].

The bioactive compound estimations in our study revealed the presence of phenolic compounds, especially *p*-coumaric acid, quercetin, and rosmarinic acid, as the major components. According to current literature, shallot bulbs contain several phenolic compounds, including apigenin, eriodictyol, gallic acid, quercetin, isoquercetin, kaempferol, catechin, and tannic acid [26]. Additionally, it has been confirmed that flavonoids and their glycosides, including quercetin and isorhamnetin, were detected in the methanolic extract of shallot bulbs [5]. Two novel furostanol saponins, named ascalonicoside A1/A2 (1a/1b) and ascalonicoside B(4), have been found in the methanolic shallot extract [1,5]. The bioactive compounds estimation of shallot extract indicated the other constituents that have not been identified in the current experimental system. Therefore, other unknown compounds in the extract required further analysis.

Plant phenolics compounds are known to possess anti-inflammatory potential, which can interact with free radicals and impede cyclooxygenase (COX), lipoxygenase, and inducible nitric oxide synthase (iNOS) [27]. The inflammation in hair follicles is triggered by oxidative stress and androgens [27,28]. Wolf et al. reported that the excessive NO production and the expression of iNOS in hHFDPC were induced by DHT [10]. It has been reported that *p*-coumaric acid possessed the anti-inflammation activity due to suppression of the nuclear factor kappa B in LPS-stimulated RAW 264.7 macrophage cells [29]. The protein expression of the pro-inflammatory enzymes (COX and iNOS) and NO production were inhibited by rosmarinic acid and quercetin [30,31,32]. Shallot extract in this study that contained those compounds exhibited anti-inflammatory potential by attenuating NO production and reducing inflammatory-induced perifollicular damage in AGA. 

Androgens affect the function of human skin, including wound healing, development of sebaceous glands, and hair growth [8]. Testosterone can be catalyzed into DHT by steroid 5α-reductases [12]. In hair follicles of AGA, DHT binds the androgen receptors, leading to hair follicle miniaturization and diminishing the period of the anagen phase [14,23]. The activities of steroid 5α-reductase type 1 and 2 in balding hair follicles were higher than in non-balding hair follicles [33]. Furthermore, the expressions of gene encoding steroid 5α-reductases (*SRD5A1*, *SRD5A2*, and *SRD5A3*) were found to upregulate in the androgen-sensitive hair follicles of AGA [34,35]. Our results postulate that shallot extract significantly attenuates the mRNA expression of *SRD5A1* and *SRD5A2*, and slightly suppresses the *SRD5A3* expression. The bioactive compounds in shallot extract may affect the regulatory elements for the expressions of *SRD5A* genes differently [36], which was not discovered in this study. So far, there is no study about the effects of bioactive compounds on the transcriptional regulatory elements associated with *SRD5A* genes and AGA. Quercetin has been reported to possess anti-inflammatory activity and anti-androgen activity through the inhibition of steroid 5α-reductases and the downregulation of androgen receptors [37,38,39,40]. Quercetin-rich extracts including *Ginkgo biloba*, *Camelia sinensis*, and *Cuscuta reflexa* exhibited promising hair growth-promoting activities via the attenuation of steroid 5α-reductases [41]. In addition, rosmarinic acid and ursolic acid, the major components of *Rosmarinus officinalis*, showed inhibitory activity towards steroid 5α-reductases [42]. It is suggested that shallot extract could reverse androgen-induced alopecia by suppressing the expressions of *SRD5A* and contributing to reducing its translation.

The Wnt/β-catenin signaling pathway is a dominant pathway that involves the development of hair follicles and sebaceous glands [11,43]. Moreover, this pathway mediates the initiation and maintenance of the anagen phase [9]. It has been proposed that there might be crosstalk between the Wnt/β-catenin and androgen pathways [44]. The differentiation of hair follicle stem cells was abolished by DHT [45]. Dickkopf 1 (DKK-1), a Wnt antagonist, promoted the premature onset of catagen and cell apoptosis [46]. A previous study demonstrated that hHFDPC secreted DKK-1 in response to DHT [47]. 

Furthermore, DHT induced the downregulation of β-catenin in hHFDPC, suggesting the androgen-induced inhibition of the Wnt/β-catenin signaling pathway [45]. β-catenin, encoded by *CTNNB1*, is known to induce the transition from telogen to anagen, leading to hair regrowth and a new hair cycle [48]. The disruption of *CTNNB1* expression contributed to abnormal hair growth in mice [49]. Recent studies have reported that quercetin increased the expression of Wnt and β-catenin [50,51]. Our results indicate that shallot extract in this study notably upregulated the expression of *CTNNB1*, leading to the elevation of the translation of β-catenin. The accumulation of β-catenin may prolong the growing phases of the anagen hair cycle, providing the promotion of hair growth [15].

Likewise, the sonic hedgehog signaling pathway regulates hair growth and hair follicle development [44,52]. It has been presumed that sonic hedgehog signaling is the downstream pathway of the Wnt/β-catenin signaling to regulate hair follicle induction [53]. The sonic hedgehog signaling is initiated by the interaction of Shh to its receptor called Patched. Consequently, Smo is dissociated from the inhibition of Patched and activates the downstream transcription factors of Gli1 [54]. This signal contributes to hair growth activation by inducing telogen-to-anagen transition during the hair follicle cycle [48]. The absence of Shh and Smo implicated the impairments of growth and morphogenesis of hair follicles [16]. It has been demonstrated that a small molecule agonist of the sonic hedgehog pathway enhanced hair growth and promoted the anagen phase in mice through the activation of gene expressions of *SHH* and *GIL1* [55]. Furthermore, the retardation of *SHH* influenced hair follicle morphogenesis and hair growth [56]. Previous studies indicated that *Polygonum multiflorum* and *Thujae occidentalis*, which are the sources of quercetin and coumarins, induced hair growth through the upregulation of Shh and β-catenin [48,57,58,59]. Moreover, our findings revealed that shallot extract activated the expressions of *SHH*, *SMO*, and *GIL1* genes in hHFDPC. These effects may lead to hair growth-promoting activity of shallot extract and the induction of telogen to the anagen phase in hair follicles.

VEGF is the important mediator that regulates blood vessel formation, wound healing, and hair growth [15,60]. Perifollicular vascularization is extensively active in the anagen phase and correlated with the upregulation of VEGF in follicular keratinocytes, leading to the acceleration of hair regrowth [61]. The size of hair follicles and the diameter of the hair shaft were also increased due to VEGF [62]. In addition, the mechanisms of minoxidil involved the upregulation of *VEGF* and its receptor in hHFDPC, leading to the promotion of angiogenesis in the anagen phase [13]. The expression levels of *VEGF* in both male and AGA were significantly lower than control without AGA [63]. Rosmarinic acid in shallot extract could enhance the protein expression of VEGF [64]. Recently, quercetin has exhibited wound healing potential by enhancing the VEGF level [50]. In our study, the expression of *VEGF* was slightly upregulated by shallot extract. This may help to promote the angiogenesis around hair follicles and stimulate hair growth.

The major constituents of shallot extract were phenolic compounds, especially quercetin, *p*-coumaric acid, and rosmarinic acid. Our findings also found that shallot extract exhibited the anti-inflammation and the modulation of genes associated with androgen, Wnt/β-catenin, sonic hedgehog, and VEGF signaling pathways. Targeting these biochemical signaling pathways of hair growth regulation would benefit AGA, a multifactorial disorder. Synergistic activities of phenolic components might contribute to shallot extract’s hair growth-promoting activities. However, further studies are required to elucidate the other bioactive compounds in shallot extract and their effects on specific signaling pathways. In the present study, an exploration of the hair growth-promoting activities of shallot extract was undertaken. Shallot extract could be applied for the development of nutraceuticals, nutricosmetics, and cosmeceuticals for AGA.

## 4. Materials and Methods

### 4.1. Preparation of Extract

Shallot (*Allium ascalonicum* L.) was purchased from the local market (Chiang Mai, Thailand) on 10 February 2021, and authenticated by the Pharmaceutical and Natural Products Research and Development Unit, Faculty of Pharmacy, Chiang Mai University (reference specimens no. PNPRDU63027). Two kilograms of shallot bulbs were blended by a food blender into a paste and macerated in methanol (ratio of solid/solvent: 1:2) for 24 h [4,5]. Then, the extract solution was filtered through Whatman filter paper no. 4 and no. 1. The clear solution was concentrated and evaporated at 50 °C by an evaporator (Hei-VAP value, Heidolph, Schwabach, Germany) until it was completely dried. Samples were kept at 4 °C for further analysis.

### 4.2. Phytochemical Estimations

#### 4.2.1. Total Phenolic Content

The Folin–Ciocalteu colorimetric method was used to determine total phenolic content. The reaction consists of the Folin–Ciocalteu reagent, sodium carbonate, and the phenolic compounds in the extract. The concentrations of standard gallic acid in the range of 0.01 to 0.2 mg/mL and their absorbances were used to plot standard curves. The results are expressed as milligrams of gallic acid equivalents per gram of extract (mg GAE/g extract) [65].

#### 4.2.2. Total Flavonoid Content

The aluminum chloride colorimetric method was employed to estimate the total flavonoid content in the sample. Aluminum chloride reacts with the C-4 keto group and either the C-3 or C-5 hydroxyl group of flavones and flavonols, resulting in a stable-colored complex. Different concentrations of (−)-epigallocatechin gallate (EGCG) in the range of 0.01 to 0.3 mg/mL and their absorbances were plotted to create the standard curve. The results are expressed in terms of milligrams of EGCG equivalents per gram of extract (mg EGCGE/g extract) [65].

#### 4.2.3. Total Polysaccharide Content

The anthrone-sulfuric acid method was conducted to quantify total polysaccharide content. The reaction of anthrone and the extract in acidic conditions was performed at 100 °C, resulting in blue-green solutions. The absorbances of D-glucose in various concentrations from 0.01 to 0.6 mg/mL were used to generate the calibration curve. The results were milligrams of D-glucose equivalents per gram of extract (mg D-glucose/g extract) [32].

#### 4.2.4. Total Protein Content

Total protein content was estimated by the Lowry method. The Folin–Ciocalteu reagent was used to interact with the cuprous ions and the side chains of tyrosine, tryptophan, and cysteine in the sample, and afterward, a blue-green color was produced. The standard protein was bovine serum albumin (BSA) with the concentration range of 0.01 to 2 mg/mL. The results are expressed as milligrams of BSA equivalents per gram of extract (mg BSA/g) [32].

### 4.3. Determination of Phenolic Compounds by Liquid Chromatography–Mass Spectrometry (LC-MS)

In accordance with the procedure of Arjin et al. [66], the samples were dissolved in 0.01% formic acid and ethanol (1:1, *v/v*) to achieve a final concentration of 1 mg/mL, and then purified with the QuEChERS dispersive SPE kit, fat + pigments (Agilent Technology, Santa Clara, CA, USA) prior to being filtered through a 0.22 µm membrane. The phenolic compounds of extract were quantified and analyzed in liquid chromatography (Agilent 1260 Infinity II series), equipped with an electrospray ion quadrupole mass spectrometer 6130 (Agilent Tech., Santa Clara, CA, USA), according to the reported method [39,67]. Solvent A was 5% formic acid. Solvent B was 5% formic acid in 10% water and 85% acetonitrile. The gradient elution was programmed as follows: 80% A at 0–8 min, 80% to 25% A at 8–24 min, 25% A at 24–28 min, 25% to 70% A at 28–34 min, 70% to 80% A at 34–36 min, and 80% A at 36–45 min. The injection volume was 5 μL. For chromatographic separation, a Restek Ultra C18 reversed-phase column (250 × 4.6 mm, 5 µm, Restek Corporation, Bellefonte, PA, USA) was used. The column oven temperature and flow rates were 30 °C and 0.5 mL/min. For mass spectrometry, the negative selected ion monitoring was implemented. Nitrogen gas was used as desolvation gas with a flow rate of 12 L/min and nebulizer pressure of 60 psi. Other parameters were programmed: a capillary voltage of −3 kV, a gas temperature °C, a fragmentation voltage of 70 V, and the full scan spectra from 100 to 1200 m/z with an acquisition rate of 250 ms/spectrum. Data acquisition and integration were processed with OpenLab software (Agilent Tech., Santa Clara, CA, USA).

### 4.4. Cell Viability Assay

Human hair follicle dermal papilla cells (hHFDPC: Promo Cell GmbH, Heidelberg, Germany) were grown in Follicle Dermal Papilla Cell Growth Medium Kit (cat no. C-26501) supplemented with 1% antibiotic-antimycotic 100× solution (Gibco™, cat no. 15240062). RAW 264.7 macrophage cells and DU-145 human prostate cancer cells were obtained from the American Type Culture Collection (Rockville, MD, USA). DU-145 cells were cultured in Roswell Park Memorial Institute 1640 medium (RPMI-1640; cat no. 31800022) containing 10% fetal bovine serum (FBS; cat no. 16000044) and 1% antibiotic-antimycotic 100× solution. RAW 264.7 macrophage cells were grown in Dulbecco’s Modified Eagle Medium (DMEM; cat no. 31600083) supplemented with 10% FBS and 1% antibiotic-antimycotic 100× solution. Cells were incubated at 37 °C in a 5% CO_2_ humidified atmosphere.

The sulforhodamine B (SRB) assay was used to determine the cytotoxic potential of shallot extract and standard reference compounds (diclofenac sodium (DF), finasteride, dutasteride, purmorphamine, and minoxidil) in a concentration range from 0.02 to 2.5 mg/mL [21]. Briefly, cells were seeded in 96-well plates (10^4^ cells/well) and incubated for 24 h. The monolayer cells were washed and treated with the tested samples (0.02–1 mg/mL). After 30 h of incubation, cultured cells were fixed on plates, and washed, dried, and stained with the SRB solution (Sigma Chemical, St. Louis, MO, USA). Tris-EDTA buffer was added to solubilize the dye extracted from stained cells. The optical density (OD) was acquired by a microplate reader (EZ Read 400, Biochrom, Cambridge, UK) at 515 nm. The highest concentration providing the percentages of cell viability above 80% was considered as non-cytotoxicity and was selected for further experiments. The percentage of cell viability was calculated by Equation (1):

(1) $$\mathrm{Cell}\;\mathrm{viability}\;\left(\%\right)=\left(\frac{{\mathrm{OD}}_{\mathrm{sample}}\;{\text{–}\;\mathrm{OD}}_{\mathrm{blank}}}{{\mathrm{OD}}_{\mathrm{control}}\;{\text{–}\;\mathrm{OD}}_{\mathrm{blank}}}\right)\text{×}\;100$$

### 4.5. Anti-Inflammatory Activity 

The Griess reaction colorimetric assay kit (Invitrogen, Thermo Fisher Scientific, Inc., Eugene, OR, USA) was used to determine the nitric oxide (NO) level in the culture medium [22]. The quantification of NO was indirectly estimated by measuring nitrite, which is the final inert product of NO. Briefly, RAW 264.7 macrophage cells were seeded into 96-well plates (10^4^ cells/well) and incubated for 24 h. The cells were pretreated with 0.1 mg/mL of diclofenac sodium (DF), 0.1 mg/mL of shallot extract, and solvent (blank). After pretreatment for 2 h, cells were incubated with and without lipopolysaccharides (LPS: Sigma Chemical, St. Louis, MO, USA). After incubation for 24 h, 150 μL of each supernatant solution was reacted with 20 μL of Griess reagent mixture and incubated for 30 min at room temperature. Then, the absorbance was read at 570 nm. The standard curve equation of reference standard sodium nitrite was used to calculate the nitrite concentration.

### 4.6. Semi-Quantitative Reverse Transcription and Polymerase Chain Reaction

DU-145 cells were used to study the expressions of genes in the androgen pathway. hHFDPC were utilized for the other remaining experiments [68,69]. Shallot extract was compared to the reference standard compounds (finasteride, dutasteride, purmorphamine, and minoxidil) at the same concentration of 0.1 mg/mL. Total RNA was isolated from cells using the E.Z.N.A.^®^ Total RNA Kit I (Omega Bio-Tek, Norcross, GA, USA), according to the manufacturer’s instructions. Qubit™ 4 fluorometer (Invitrogen, Carlsbad, CA, USA) and Qubit™ RNA HS Assay Kit (Invitrogen, Carlsbad, CA, USA) were used to determine the concentration of the purified RNA. The RNA solution was maintained at −20 °C until it was used. Gene expression levels were carried out by the semi-quantitative RT-PCR [70]. Complementary DNA was synthesized using the MyTaq™ One-Step RT-PCR Kit (Bioline, Memphis, TN, USA). Primer sequences used are as follows: *SRD5A1*: AGCCATTGTGCAGTGTATGC and AGCCTCCCCTTGGTATTTTG; *SRD5A2*: TGAATACCCTGATGGGTGG and CAAGCCACCTTGTGGAATC; *SRD5A3*: TCCTTCTTTGCCCAAACATC and TCCTTCTTTGCCCAAACATC; *SHH*: AAAAGCTGACCCCTTTAGCC and GCTCCGGTGTTTTCTTCATC; *SMO*: GAAGTGCCCTTGGTTCGGACA and CCGCCAGTCAGCCACGAAT; *GIL1*: GCAGGGAGTGCAGCCAATACAG and GAGCGGCGGCTGACAGTATA; *CTNNB1*: CCCACTAATGTCCAGCGTTT and AACCAAGCATTTTCACCAGG; *VEGF*: CTACCTCCACCATGCCAAGT and GCGAGTCTGTGTTTTTGCAG; *GAPDH*: GGAAGGTGAAGGTCGGAGTC and CTCAGCCTTGACGGTGCCATG.

Agarose gel electrophoresis was performed to detect the RT-PCR products [70]. The gel images and band intensity were acquired by the Gel Doc™ EZ System (Version 3.0; Bio-Rad) and Image Lab™ software (Bio-Rad). The expression of target genes was normalized by the *GAPDH* expression value and expressed as the relative expression value. Each sample was analyzed in triplicate.

### 4.7. Statistical Analysis

All the tests were conducted in triplicates. Results are expressed as a mean ± standard error of the mean. Statistical comparisons were performed using the Jamovi version 1.6.23 (The Jamovi Project, Sydney, Australia). One-way analysis of variance followed by Tukey’s test was used to determine the statistical differences between the mean of pairs. *p*-value < 0.05 was considered significant.

## 5. Conclusions

Shallot is one of the most essential horticultural ingredients in Asian cuisine and is used as a traditional medicine for hair loss. Our findings show that shallot extract contains phenolic compounds, namely, quercetin, rosmarinic, and *p*-coumaric acids, contributing to its anti-inflammation potential via NO inhibition. Interestingly, the gene expressions of *SRD5A2* were downregulated by shallot extract and comparable to standard drugs (finasteride and dutasteride), leading to the reduction in androgenic effects on androgen-sensitive hair follicles. On the other hand, shallot extract enhanced the expressions of *CTNNB1*, *SHH*, *SMO*, *GIL1*, and *VEGF*, providing hair growth promotion effects via the maintenance of the anagen phase and the improvement of blood flow in hair follicles. In summary, shallot extract could promote hair growth by anti-inflammation and regulations of genes in androgen, sonic hedgehog, Wnt/β-catenin, and angiogenesis signaling pathways. The results of this study provide a sufficient basis for the utilization of shallot extract, which could be further developed as nutraceuticals, nutricosmetics, and cosmeceuticals for promoting hair growth.

## Figures and Tables

**Table 1:** Content of bioactive compounds of shallot extract.

Bioactive Compounds	Content	
Total polysaccharide content	0.90 ± 0.06	mg D-glucose/g
Total protein content	1.01 ± 0.04	mg BSAE/g
Total phenolic content	4.69 ± 0.42	mg GAE/g
Total flavonoid content	<0.003	mg EGCGE/g
Phenolic compounds		
*p*-Coumaric acid	1.091 ± 0.011	mg/g
Quercetin	0.029 ± 0.002	mg/g
Rosmarinic acid	0.234 ± 0.007	mg/g

Note: Milligrams of gallic acid equivalents per gram of extract (mg GAE/g extract); milligrams of epigallocatechin gallate equivalents per gram of extract (mg EGCGE/g extract); milligrams of D-glucose equivalents per gram of extract (mg D-glucose/g extract); milligrams of bovine serum albumin equivalents per gram of extract (mg BSAE/g extract).

## Data Availability

Not applicable.
